# The influence of immigrant background and parental education on overweight and obesity in 8-year-old children in Norway

**DOI:** 10.1186/s12889-023-16571-1

**Published:** 2023-08-29

**Authors:** B. Øvrebø, M. Kjøllesdal, T. H. Stea, A. K. Wills, E. Bere, P. Magnus, P. B. Juliusson, I. H. Bergh

**Affiliations:** 1 Department of Health and Inequalities, Norwegian Institute of Public Health, Oslo, Norway; 2 Centre for Evaluation of Public Health Measures, Norwegian Institute of Public Health, Oslo, Norway; 3 Department of Public Health Science, Norwegian University of Life Sciences, Ås, Norway; 4 Department of Health and Nursing Sciences, University of Agder, Kristiansand, Norway; 5 Department of Nutrition and Public Health, University of Agder, Kristiansand, Norway; 6 Faculty of Health Sciences, University of Bristol, Bristol, UK; 7 Department of Sport Science and Physical Education, University of Agder, Kristiansand, Norway; 8 Centre for Fertility and Health, Norwegian Institute of Public Health, Oslo, Norway; 9 Department of Health Registry Research and Development, Norwegian Institute of Public Health, Bergen, Norway; 10 Department of Clinical Science, University of Bergen, Bergen, Norway; 11 Children and Youth Clinic, Haukeland University Hospital, Bergen, Norway

**Keywords:** Prevalence, Overweight, Obesity, Immigrant, Parental education, Children

## Abstract

**Background:**

Little is known about the prevalence of overweight/obesity and socio-economic position (SEP) in children with immigrant background in Scandinavia. The purpose of this study is to examine the prevalence of overweight/obesity by immigrant background among children in Norway and to explore the role of SEP in explaining differences in weight status.

**Methods:**

Anthropometric data from 8,858 children (age 8.3 years) from the population-based Norwegian Childhood Growth Study were used. Information about immigrant background, country of origin, and parental education (used as an indicator of SEP) were provided by Statistics Norway. For children with immigrant background, regional background was determined based on country of origin. Prevalence ratios (PR) were estimated for overweight/obesity and weight-to-height-ratio (WHtR) ≥ 0.5 by immigration and regional background, using generalized estimating equation log-binominal models adjusting for sex, age, survey year (model 1), residential area, population density (model 2) and parental education (model 3).

**Results:**

Children with immigrant background had a higher prevalence of overweight/obesity and WHtR ≥ 0.5 than non-immigrant background children. Adjusted for parental education, children with an immigrant background from Southern and Eastern Europe, Asia except South-Asia, and Africa had a higher prevalence of overweight/obesity [PR: 1.37 (95% confidence interval (CI): 1.10–1.72), 1.28 (1.05–1.57), 1.47 (1.13–1.91), respectively] than children with a non-immigrant background. Children originating from Asia except South-Asia had a higher prevalence of WHtR ≥ 0.5 (PR: 1.64, CI: 1.25–2.15) compared to non-immigrant background children. The adjustment for parental education did not substantially change the results.

**Conclusion:**

Children with immigrant background had higher prevalence of overweight/obesity than non-immigrant background children. The difference varied according to region of origin but not substantially according to parental education. There is a need for culturally acceptable preventative measures targeting the parents of immigrant background children.

**Supplementary Information:**

The online version contains supplementary material available at 10.1186/s12889-023-16571-1.

## Background

Obesity is an important risk factor for non-communicable diseases, and one of the largest public health challenges of present time [1]. Childhood obesity is associated with adverse physiological and psychological health [2, 3] and is a predictor for obesity and risk for related chronic diseases in adulthood [4, 5]. In some high-income countries overweight and obesity rates have stabilized [6]. However, a stabilization among children from the general population may mask differences in overweight and obesity between sub-groups, as evidence suggest increasing differences in overweight and obesity by both socio-economic position (SEP) and immigrant background [7–9].

Several studies from Europe, including Scandinavia, show a higher prevalence of overweight and obesity among children with immigrant background compared to those with non-immigrant background [10–17]. A recently published, comprehensive Norwegian study using register-based data from the specialist health care services, showed that a higher proportion of children with immigrant parents than children with Norwegian-born parents were treated for obesity [18]. However, only a few Norwegian studies have examined weight status among children with immigrant background, and the implications from these studies are limited due to lack of recently collected data [19], small samples, and a limited age range [15]. Several studies have indicated that weight status varies according to ethnicity and geographical region, but more knowledge about differences in overweight/obesity prevalence among immigrants according to region of origin is needed [11, 13, 20].

In high-income countries, overweight and obesity is generally more common among children of lower SEP [6, 21], and in Scandinavia, immigrants are overrepresented in socially disadvantaged groups [22]. A systematic review of Scandinavian studies reported that the association between SEP and various health outcomes varies according to immigrant background [20]. However, existing studies do not support that body mass index (BMI) and overweight/obesity vary by parental education level among children with immigrant background [15, 19, 23], and conflicting results have been presented from studies aiming to identify how parental education may contribute to explain differences in risk of overweight and obesity between children with and without immigrant background [13, 24]. Addressing overweight and obesity requires more knowledge about weight status among children with different regional backgrounds, while controlling for SEP [14, 20, 25].

Based on previous findings [10–17], we hypothesized a higher prevalence of overweight/obesity among children with immigrant background compared to non-immigrant background children. To test this, we used a large Norwegian population-based survey with repeated samples of 8-year-olds to examine differences in prevalence of overweight/obesity by immigrant background, and to explore whether region of origin and SEP may influence possible differences in weight status.

## Methods

We used anthropometric data from three cohorts of third graders (~ 8 years) from the Norwegian Childhood Growth Study (NCGS) and information about participants’ immigrant background, country of origin, and parental education level from Statistics Norway. Data were linked to participants through the national unique personal identification number. Information on residing area and population density was obtained from the geographical location of the schools that the children attended.

### Study design

The NCGS is a nationally representative, repeated cross-sectional study conducted by the Norwegian Institute of Public Health in collaboration with the School Health Service. We used data from the surveys in 2010, 2012, and 2015. Sampling was conducted in two steps. Firstly, ten out of 19 counties were sampled from five regional strata in Norway. Secondly, 130 elementary schools were randomly sampled within each county. All third graders in the participating schools were sampled. The individual-level participation rate was > 80%. Further details about sampling design and surveys are available elsewhere [26].

### Participants

Information about immigrant background, height, and weight, were available from 9,991 of the 10,024 eligible third graders.

Children were categorized into two main groups: 1) *non-immigrant background*, consisting of children born in Norway (or abroad) to two Norwegian-born parents; and 2) *immigrant background*, consisting of children with two immigrant parents. Children with immigrant background could be born in Norway or abroad. To clearly separate the non-immigrant and immigrant background groups, children with one foreign-born and one Norwegian-born parent were excluded (*n* = 1,112).

**Fig. 1:**
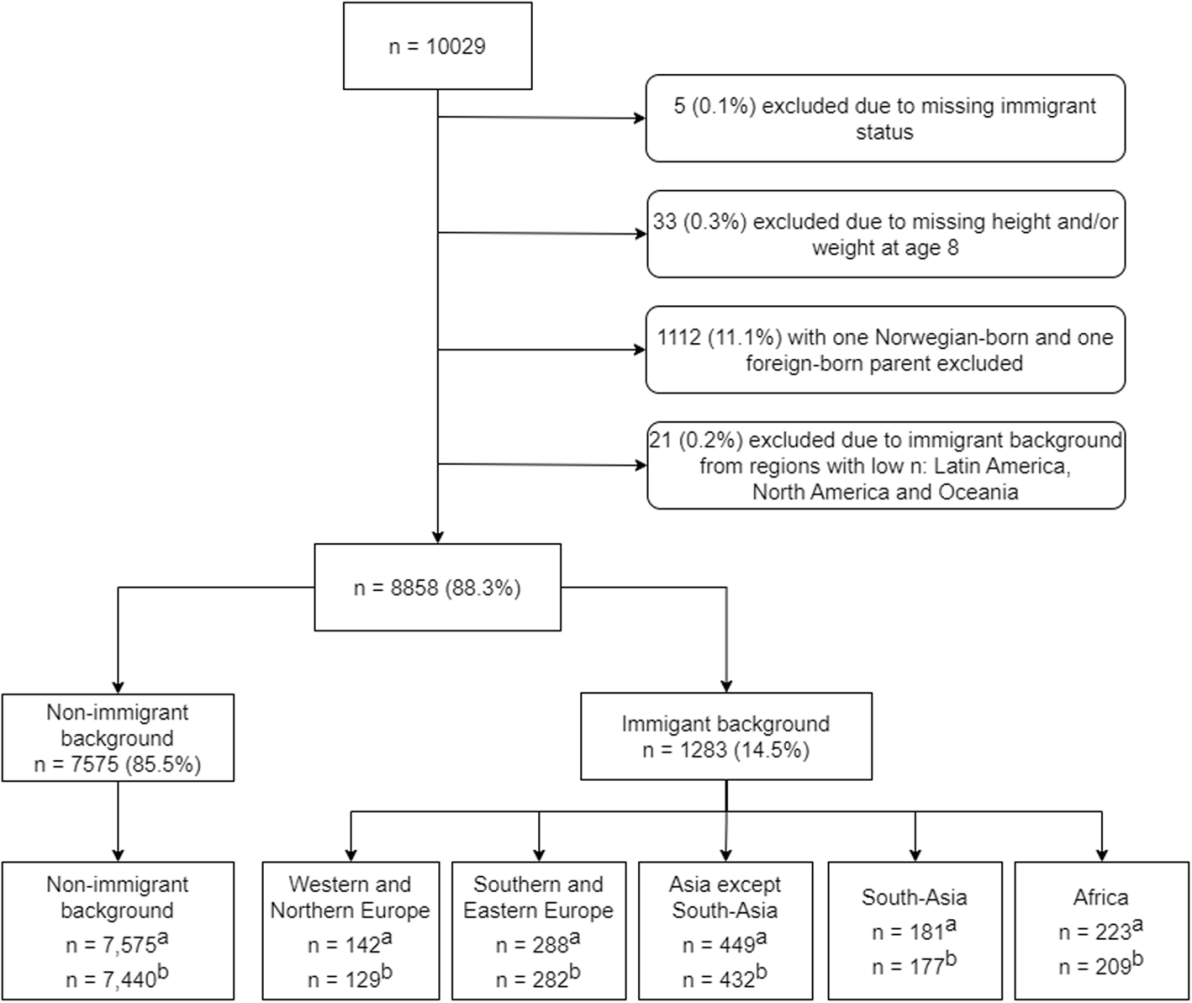
Flow chart of sample in the current study ^a^ n included in the total sample and descriptives. ^b^ n included in the main analysis with complete cases on all covariates

Children with immigrant background were further categorized into region of origin based on their own country of birth or if Norwegian-born to immigrant parents the mother's country of birth. The regions of origin groups were 1) Western and Northern Europe; 2) Southern and Eastern Europe; 3) Asia except South-Asia; 4) South-Asia; and 5) Africa. The following regions were excluded due to few participants (Latin America, North America (USA and Canada), and Oceania, total *n* = 21), leaving a sample of 8,858 children (Fig. 1). The regions of origin were defined based on a combination of classification systems from United Nations Geographic Region [27], Statistics Norway [28], and the World Health Organization Western Pacific Region [29] to reflect both standard divisions of regions and known differences in overweight/obesity prevalence between regions (details in Supplementary Text 1, Additional file 1).

### Overweight and obesity

Height, weight, and waist circumference were measured during the fall semester of grade 3 by the school health nurses. Additional details about the data collection, equipment, data entering, and quality assurance processes have been described elsewhere [26, 30].

Weight status was calculated by age- and sex-specific BMI cut-off-values based on the International Obesity Task Force (IOTF) criteria [31], and waist-to-height-ratio (WHtR) by waist circumference in cm/height in cm. The IOTF cut-offs correspond to the adult BMI-definitions for overweight and obesity. In the main analyses binary outcomes were used: overweight/obesity (BMI ≥ IOTF overweight cut-off) and WHtR ≥ 0.5.

### Parental education

Parental education was used as an indicator for SEP. Highest parental education level by either mother or father, attained at the year of measurement of the child, was collapsed into three levels: primary (primary education or less), secondary (lower and upper secondary education including post-secondary non-tertiary education), and higher education (education in university/college).

### Residential area and population density

The residential areas in the sample were Northern, Central, Western, and Southern/Eastern Norway. Population density was categorized as urban (municipalities with a population > 50,000), semi-urban (population between 15,000 and 50,000), and rural (population < 15,000) in accordance with the classification established by Statistics Norway.

### Statistical analyses

Since prevalences of overweight and obesity were relatively stable over the years (2010 to 2015) of the three surveys [32], data were pooled to provide a larger sample size. Age, sex, residential area, population density, and parental education were presented for the total sample, by immigrant and regional background.

The crude prevalences of overweight including obesity and WHtR ≥ 0.5 were estimated for non-immigrant and immigrant background children in total and by region of origin. Prevalences were presented by sex and parental education in total and by region of origin within each group. X^2^-tests or Fishers Exact tests were used to investigate differences between sexes and parental education levels within each group. The prevalences in each IOTF BMI category are shown in Supplementary Table 1, Additional file 2.

### Main analysis

Generalized estimating equation (GEE) log-binominal models were fitted to account for clustering in the data and used to test differences in overweight/obesity by immigrant background. Schools were included as a cluster variable. Prevalence ratios (PR) with 95% confidence intervals (CI) for the outcomes were estimated, with the children’s immigrant background as the exposure and children with non-immigrant background as the reference group. For common outcomes (> 10%), PR is preferred to the prevalence odds ratios (PORs) as the latter may exaggerate the true relative prevalence [33, 34]. Additionally, interpreting PRs are more intuitive than interpretation of PORs [35, 36].

Three sets of models were estimated to adjust for socio-demographic factors associated with overweight/obesity in Norwegian children [26, 37]; sex, age, and survey year were adjusted for in model 1, residential area and population density were added in model 2, and parental education (three categories) were additionally added in model 3 to investigate the influence of SEP. Models were conducted using participants with complete cases for all covariates included in model 3 (*n* = 8,669; *n* = 189 excluded, Fig. 1). Stata version 16.0 was used for all analyses.

### Sensitivity analysis

Sensitivity analysis among children originating from South-Asia were conducted using the IOTF cut-offs corresponding to the lower cut-offs for adult overweight (BMI ≥ 23 kg/m^2^) and obesity (BMI ≥ 27 kg/m^2^) suggested by the World Health Organization for individuals in the South-Asian population [29, 38].

To investigate if the results were robust to the choice of statistical model, the models were re-estimated using logistic regression, as we acknowledge that log-binominal models may overestimate precision [39, 40].

To further investigate weight outcomes among children with immigrant background, supplemental analysis on differences between foreign- versus Norwegian-born children with immigrant background were conducted.

### Ethics

Data are from the NCGS, managed by the Norwegian Growth Cohort. The NCGS surveys were conducted in accordance with the Helsinki Declaration and the research approved by the Regional Committee of Medical and Health Research Ethics (2010/938) and the Norwegian Data Inspectorate. Detailed information about the NCGS was sent to parents or guardians, and the School Health Service obtained written informed consent from parents or other legal guardians on behalf of the Norwegian Institute of Public Health prior to each survey.

## Results

### Descriptive characteristics of study sample

Approximately 15% of participants had an immigrant background, of which the majority (61%) were Norwegian born to immigrant parents (Supplementary Table 2, Additional file 3).

The proportion of girls and distribution of age were similar between children with immigrant- and non-immigrant background (Table 1). A higher proportion of children with immigrant background attended schools in the South-Eastern area of Norway, and in urban areas, than non-immigrant background children (71% vs. 51% and 88% vs. 75%, respectively) with some variation by region of origin groups.

**Table 1:** Characteristics of study sample, in total and by immigrant and regional background (*n* = 8858)

	All	Non-immigrant background	Immigrant background, total	Immigrant background, by region of origin				
				Western and Northern Europe	Southern and Eastern Europe	Asia except South-Asia	South-Asia	Africa
% (N)	100 (8858)	85.5 (7575)	14.5 (1283)	1.6 (142)	3.2 (288)	5.1 (449)	2.0 (181)	2.5 (223)
Age, mean (SD)	8.3 (0.3)	8.3 (0.3)	8.3 (0.3)	8.3 (0.3)	8.3 (0.3)	8.3 (0.3)	8.3 (0.3)	8.4 (0.4)
Sex, % (n)								
Boys	51.3 (4542)	51.8 (3927)	47.9 (615)	58.5 (83)	47.9 (138)	46.3 (208)	51.4 (93)	41.7 (93)
Girls	48.7 (4316)	48.2 (3648)	52.1 (668)	41.6 (59)	52.1 (150)	53.7 (241)	48.6 (88)	58.3 (130)
Residing area, % (n)								
South-East	53.7 (4677)	50.8 (3779)	70.5 (898)	51.4 (72)	62.4 (179)	76.7 (343)	85.0 (153)	68.6 (151)
West	23.0 (2004)	23.5 (1,749)	20.0 (255)	27.1 (38)	27.9 (80)	15.9 (71)	12.2 (22)	20.0 (44)
Central	11.7 (1016)	12.8 (953)	5.0 (63)	10.0 (14)	4.5 (13)	5.4 (24)	2.2 (4)	3.6 (8)
North	11.7 (1019)	12.9 (961)	4.6 (58)	11.4 (16)	5.2 (15)	2.0 (9)	0.6 (1)	7.7 (17)
Population density, % (n)								
Urban	77.0 (6713)	75.2 (5594)	87.8 (1119)	71.4 (100)	84.0 (241)	90.2 (403)	97.8 (176)	90.5 (199)
Semi-urban	14.4 (1257)	15.6 (1163)	7.4 (94)	14.3 (20)	11.2 (32)	5.2 (23)	1.7 (3)	7.3 (16)
Rural	8.6 (746)	9.2 (685)	4.8 (61)	14.3 (20)	4.9 (14)	4.7 (21)	0.6 (1)	2.3 (5)
Parental education^a^, % (n)								
Primary	7.5 (658)	4.0 (301)	28.8 (357)	9.9 (13)	15.9 (45)	32.0 (139)	36.0 (64)	45.3 (96)
Secondary	33.6 (2961)	33.7 (2548)	33.4 (413)	22.9 (30)	42.8 (121)	33.9 (147)	37.1 (66)	23.1 (49)
Higher	58.9 (5192)	62.4 (4724)	37.8 (468)	67.2 (88)	41.3 (117)	34.1 (148)	27.0 (48)	31.6 (67)

*N* number, *SD* Standard deviation

^a^Highest parental education level by either mother or father attained the year of measurement: primary (primary education or less), secondary (lower and upper secondary education), and higher education (education in university/college)

A higher proportion of immigrant background children had primary parental education level than non-immigrant background children (29% vs. 4%), with the highest proportions among those from Asia except South-Asia (32%), South-Asia (36%), and Africa (45%) (Table 1).

### Weight status descriptives according to sex and parental education within groups

**Fig. 2:**
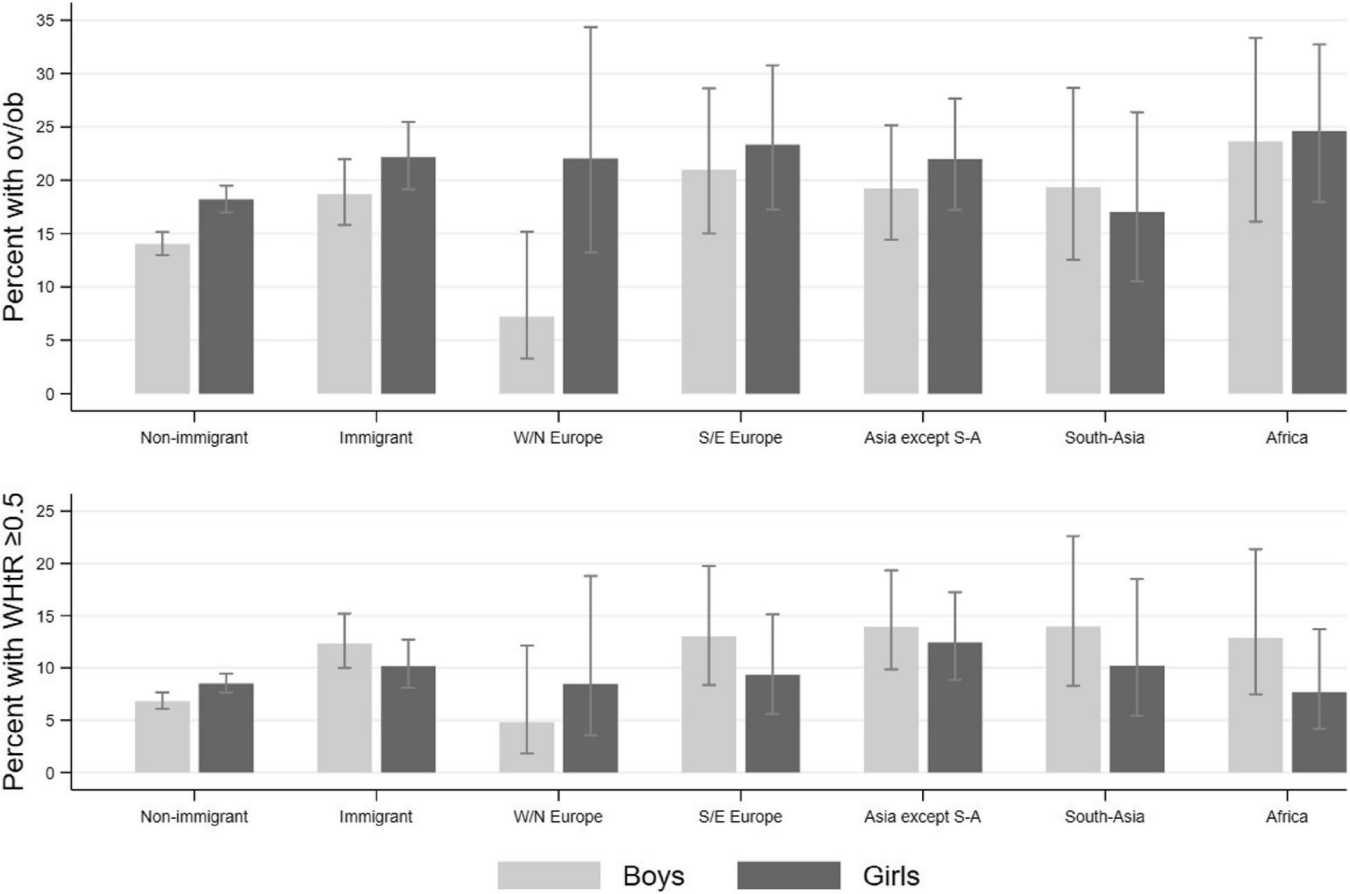
Children with overweight/obesity (top) and WHtR ≥ 0.5 (bottom) by sex within groups Proportion (with 95% confidence intervals) of children with overweight/obesity (top) and WHtR ≥ 0.5 (bottom) by sex within non-immigrant- and immigrant background in total, and groups by region of origin. Asia except S-A: Asia except South-Asia; ov/ob: overweight including obesity; S/E: Southern and Eastern; W/N: Western and Northern; WHtR: waist-to-heigh-ratio

Among children with immigrant background (total), no significant sex difference was seen for overweight/obesity or WHtR ≥ 0.5, Fig. 2 and Supplementary Table 3, Additional file 4. Among children with non-immigrant background, a higher proportion of girls than boys had overweight/obesity (18% vs 14%, *p* < 0.001) and WHtR ≥ 0.5 (9% vs. 7%, *p* = 0.006).

**Fig. 3:**
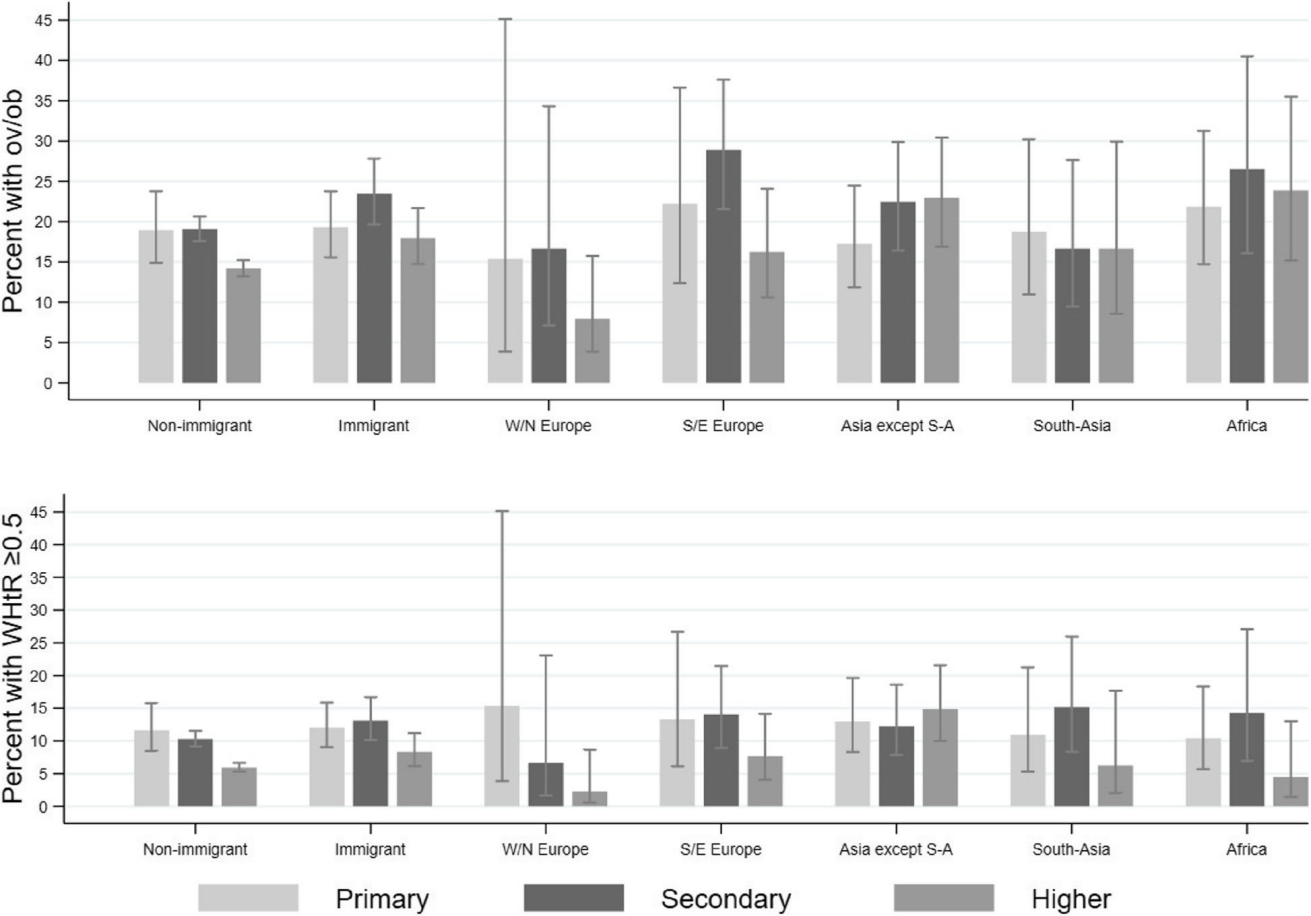
Children with overweight/obesity (top) and WHtR ≥ 0.5 (bottom) across parental education levels within groups Proportion (with 95% confidence intervals) of children with overweight/obesity (top) and WHtR ≥ 0.5 (bottom) across parental education levels within non-immigrant- and immigrant background in total, and groups by region of origin. Highest parental education level by either mother or father attained the year of measurement. Asia except S-A: Asia except South-Asia; ov/ob: overweight including obesity; S/E: Southern and Eastern; W/N: Western and Northern; WHtR: waist-to-heigh-ratio

Among children with immigrant background, no clear patterns of associations between parental education and overweight/obesity or WHtR ≥ 0.5 were observed. Among non-immigrant background children, those with low parental education had higher prevalence of overweight/obesity and WHtR ≥ 0.5 (*p*-values < 0.001) than those with higher parental education (Fig. 3 and Supplementary Table 4, Additional file 5).

### Prevalence ratios of overweight and obesity outcomes by children with immigrant background compared to children with non-immigrant background

**Fig. 4:**
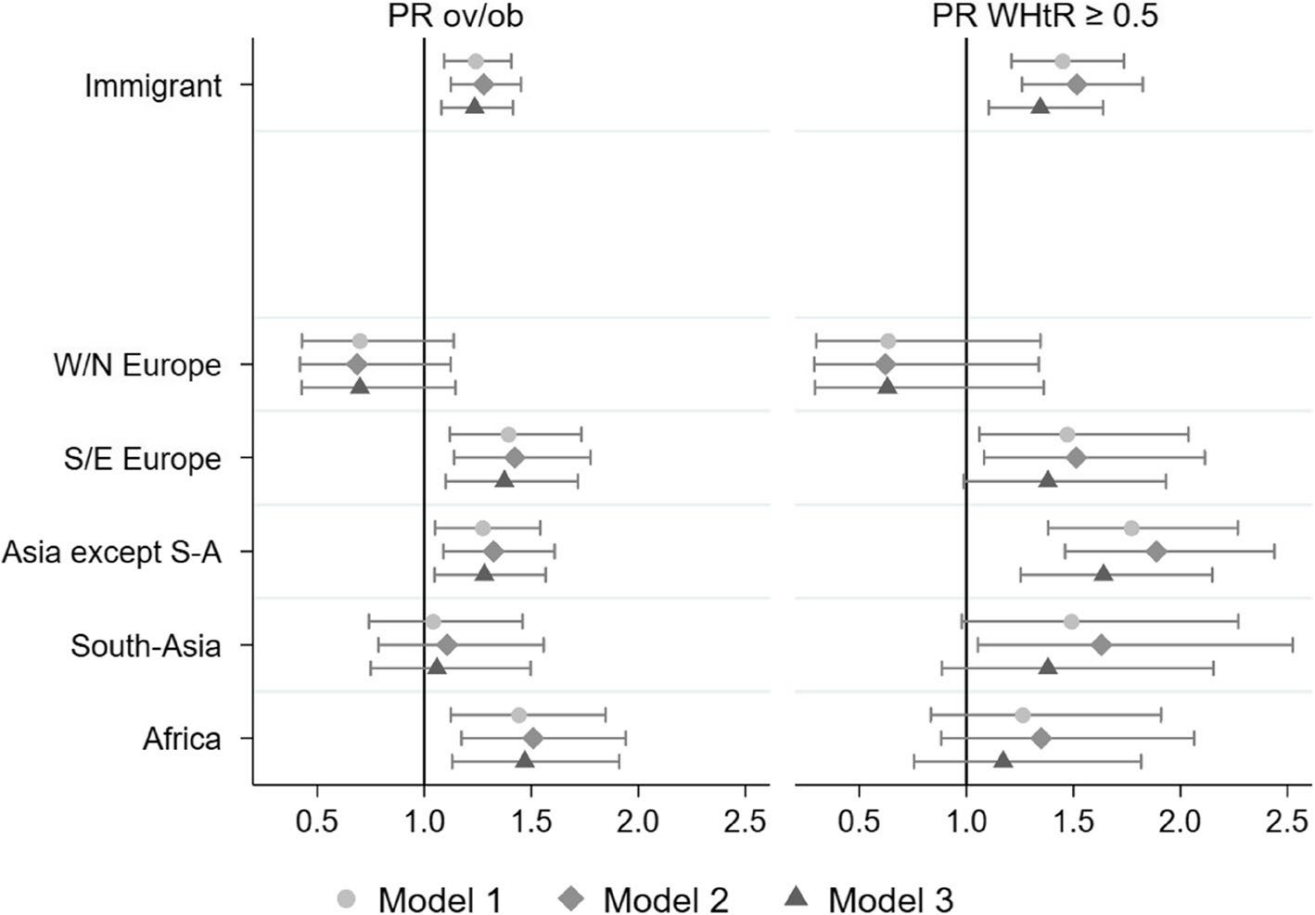
Overweight/obesity and WHtR ≥ 0.5 for immigrant background children (reference: non-immigrant background children) Prevalence ratios (PR) (95% confidence interval) of overweight/obesity (left) and WHtR ≥ 0.5 (right) for 8-year-old children in Norway by immigrant background in total and groups by region of origin with children, with non-immigrant background as the reference. Three sets of GEE log-binominal models were conducted using children with non-immigrant background as the reference category (indicated by black vertical the line). Model 1 with adjustments for age, sex, and survey year; model 2 additionally adjust for residing area and population density; and model 3 additionally adjust for parental education level. The analyses were conducted with complete cases on all covariates. Asia except S-A: Asia except South-Asia; GEE: generalized estimating equation; ov/ob: overweight including obesity; PR: Prevalence ratio; S/E: Southern and Eastern; W/N: Western and Northern; WHtR: waist-to-heigh-ratio

Adjusted for demographic factors and survey year (model 2), there was a 28% higher prevalence of overweight/obesity and a 52% higher prevalence of WHtR ≥ 0.5 among immigrant background children compared to non-immigrant background children (Fig. 4 and Supplementary Tables 5 and 6 in Additional files 6 and 7). By region of origin, the prevalence of overweight/obesity was higher among children originating from Southern/Eastern Europe (42%), Asia except South-Asia (32%) and Africa (51%), and for WHtR ≥ 0.5 among children originating from Southern/Eastern Europe (51%), Asia except South-Asia (89%) and South-Asia (63%), compared to non-immigrant background children.

Adjustment for parental education in model 3 did not substantially change the results for overweight/obesity, but attenuated the estimates for WHtR ≥ 0.5 somewhat, leaving only children orginating from Asia except South-Asia with higher prevalence compared with non-immmigrant children (details in Supplementary Tables 5 and 6, Additional files 6 and 7).

### Sensitivity analysis

Using the lower suggested cut-offs for children originating from South-Asia increased the crude proportion of children with overweight/obesity from 18 to 32% (Supplementary Table 7, Additional file 8), and the PR indicated a 97% higher prevalence of overweight/obesity compared to the non-immigrant background children (PR model 3: 1.97, 95% CI: 1.55; 2.51, *p* < 0.001), Supplementary Table 8 in Additional file 9.

Sensitivity analysis using logistic models did not alter the findings, Supplementary Table 9 in Additional file 10.

Analysis of children with immigrant background born in Norway versus those who were foreign-born showed no differences in crude prevalence of overweight/obesity or WHtR ≥ 0.5, Supplementary Table 10, Additional file 11.

## Discussion

### Key findings

Overall, children with immigrant background had a higher prevalence of overweight/obesity and WHtR ≥ 0.5 than non-immigrant background children. Children with immigrant background from Southern/Eastern Europe, Asia except South-Asia, and Africa had a higher prevalence of overweight/obesity than children with non-immigrant background, and children with background from Asia except South-Asia, had a higher prevalence of WHtR ≥ 0.5, adjusted for sociodemographic factors and parental education. Parental education did not explain a substantial part of the differences in prevalence of overweight/obesity between children with immigrant background and non-immigrant background.

### Interpretation

Our findings support the hypothesis that children with immigrant background have a higher prevalence of overweight/obesity compared to non-immigrant background children, but with variations by region of origin. While our study is not directly comparable to other studies (e.g. regarding the age of the participants, the regional or country of origin the immigrant background children had and were analyzed by, sample size and other factors included in the studies), our main findings are in line with other European and Scandinavian studies which have reported higher risk of overweight/obesity among children with immigrant background compared to reference groups [10–17].

In the present study, children with Southern/Eastern European origin represented the second largest immigrant group (Table 1) and had the second highest PR estimates for overweight/obesity. To our knowledge, information about prevalence of overweight/obesity among children with immigrant background from Europe in Norway is scarce, despite the fact that European immigrants, and in particular immigrants from Eastern Europe, have constituted the largest group of immigrants to Norway the last decade [22]. However, an Australian study also found higher prevalence of overweight/obesity in children (4–13 years) with immigrant background from South-Eastern and Eastern Europe compared to children with Australian background [41]. This is in contrast to a Swedish study which did not find higher odds of overweight/obesity among children (aged 4.8 years) with parental ethnicity from Eastern Europe compared to Swedish [13]. The Swedish study included children with Eastern European background, whereas the sample in the current study included children with both Southern and Eastern European backgrounds. This difference could be one reason for the contrasting findings as children living in Southern European countries have the highest prevalence of overweight and obesity in the WHO European region [42].

For children originating from Africa and Asia, existing Scandinavian studies have in line with our results reported an increased risk of overweight/obesity in children with similar background, despite some differences in methodology (e.g. grouping of children according to origin, children’s age, covariates) [13, 15]. Kanolkar et al. [13] included Asian participants with background from Iran and Turkey which were not highly represented countries in our Asia except South-Asia group. Still, together these findings indicate that immigrant background children in Scandinavia originating from Africa and Asia may have a higher risk of overweight/obesity.

As expected, applying the proposed lower BMI cut-off values for overweight and obesity for children with immigrant background originating from South-Asia [29, 38] increased the prevalence and PR substantially (Supplementary Tables 7 and 8, Additional files 8 and 9), similar to other findings [15]. Hence, using the general IOTF cut-offs may mask the actual overweight/obesity prevalence in immigrant background children from this part of the world.

Children with Western/Northern European origin are likely to have a cultural background and lifestyle being more similar to the children with non-immigrant background which may explain the lack of differences in weight outcomes between these groups.

In general, the PR estimates for WHtR ≥ 0.5 in our study reflected the overweight/obesity pattern for the regional groups among immigrant background children, but fewer significant findings were observed. Nevertheless, children from Asia except South-Asia, had higher prevalence of WHtR ≥ 0.5 than non-immigrant background children (model 3). Few previous studies have investigated indicators for central obesity in children with immigrant background, but Brug et al. [24] observed a higher mean waist circumference among non-natives (based on parental country of birth) compared with natives in a sample of European adolescents. Noticeably, the PR estimates of WHtR ≥ 0.5 for children with immigrant background from Asia except South-Asia, was relatively higher than the PR estimates for overweight/obesity in our study (Supplementary Tables 5 and 6, Additional files 6 and 7). This could be a concern since central obesity is more closely related to cardiovascular disease than total body fat later in life [43].

Among immigrant background children from Africa, we did not find significantly higher PR of WHtR ≥ 0.5 (any models), whereas the highest overweight/obesity PR estimate was observed among this group compared to groups with other regions of origin. Findings from our study are similar to results reported among Somali women in Norway showing a higher prevalence of overweight/obesity compared to women in Somalia while there was no difference in central obesity assessed by WHtR between the two groups [44]. Together, even though we compare children and adults, these results may indicate a different fat distribution in individuals with African background.

Our results for overweight/obesity and the influence of parental education are similar to a Swedish study [13] showing that SEP, measured by parental education and income, did not explain the differences in overweight/obesity in preschool children with different immigrant background compared to non-immigrant children. Also, an Australian study concluded that excess weight in children of immigrants was not explained by socioeconomic disadvantage [45]. However, in contrast to our findings, an older study from Germany found that mothers’ education level explained a substantial part of the difference in overweight/obesity prevalence between immigrant children and non-immigrant children attending primary school [46], which could be one explanation for the contrasting findings as we used the highest education level independent of mother or father. Regarding WHtR ≥ 0.5, we are not aware that other have investigated the role of SEP, but as for overweight/obesity parental education did not seem to explain a substantial part of the difference by immigrant background either. Moreover, the descriptive analyses from our study (Fig. 3) showed that overweight/obesity and WHtR ≥ 0.5 did not differ by parental education among children with immigrant background, only among children with non-immigrant background which correspond to Scandinavian studies reporting no association between parental education and BMI or overweight/obesity [15, 19, 23]. Immigrant parents may not have jobs, income or social position that correspond to their level of education from their country of origin, and low educated immigrant parents may be a more heterogenous group than low educated non-immigrants. Thus, parental education level may represent different challenges and different influences on weight outcomes in children with immigrant background than non-immigrant background children.

Our findings suggest that factors other than parental education level are more important in explaining the differences in weight status among immigrant children in Norway. We lack additional data that can explain why immigrant background children might have a higher prevalence of overweight/obesity than non-immigrant background children. However, other studies have reported that children born to parents outside the Nordic Region (vs. Nordic) had higher intakes of unhealthy food after adjustment for parental education level [23], and children of immigrant parents also had higher likelihood of low physical activity and having overweight/obesity compared to those with a Swedish parent [16]. Brug et al. [24] found that non-natives European adolescents had both less favorable weight status and energy balance related behaviors than native adolescents. In addition, genetic, physiological, epi-genetic, body size preferences, and acculturation (i.e. changing attitudes and behaviors under the influence of the host culture) may also explain why children with immigrant background have a higher risk of overweight/obesity than children with non-immigrant background [25].

### Strengths and limitations

The data used in the present study stem from a large national, population-based survey of objectively measured height, weight and waist circumference with a high response rate, and with linkage to register data for immigrant background, country of origin and parental education. The study provides valuable insight into prevalence of overweigt/obesity and WHtR ≥ 0.5 by immigrant background and region of origin and the influence of parental education.

Analyzing WHtR, in addition to IOTF for overweight/obesity, is an additional strength as it is an indicator of central obesity and provides information about the distribution of fat [47]. In our opinion, the sensitivity analyses using the lower IOTF cut-off values for overweight and obesity among children originating from South-Asia [29, 38] is a strength as well. However, we are aware that others regard the existing IOTF cut-offs as appropriate [48].

A limitation of the present study is that we used parental education as the sole indicator of SEP. Additional indicators, such as parental income and occupation, could have provided more information about the association between SEP and weight status [49]. Unfortunately, other indicators of SEP were not available in this dataset. Information about education attainment is also lacking for some immigrants living in Norway [50]. However, in the present study, and in line with another study [13] the parent with the highest education level (independent of mother or father) was applied in the construction of the variable. This process reduced missing values.

Another study limitation was that separate analyses reflecting overweight and obesity prevalence were not feasible due to the low number of individuals with obesity. In addition, the relatively low number of participants in groups of immigrant children reflecting different regions of origin provided high uncertainty in our estimates. Weight status in children can also vary between countries within regions, which we could not elucidate in our sample. We analyzed children with immigrant background “born in Norway” and “foreign-born” to immigrant parents as one group to keep a substantial sample size. We acknowledge that these two groups may differ with respect to weight development and weight status, e.g., due to factors not registered in the present study, such as length of stay in Norway. Still, sensitivity analyses did not show any significant differences in the crude overweight/obesity or WHtR ≥ 0.5 between the two groups (Supplementary Table 10, Additional file 11)*.* We also excluded children with one foreign-born and one Norwegian-born parent to clearly separate the immigrant- and non-immigrant background groups. This is likely a growing group that warrants further investigation to better understand the overweight/obestiy among immigrant children.

### Implications

Our results show that immigration background should be considered in planning and implementing programs to prevent childhood obesity in mixed population groups. Health workers should be aware that unhealthy weight status is more common in some groups of children with immigrant background. In Norway, regular assessments of weight and height of children from birth to age 13 are provided as part of the health examinations offered to all parents and their children in the health station and school health service. These health examinations provide an opportunity to address weight development of the child with the parents, and consult the parents about prevention of overweight and obesity. However, there may be a need for culturally relevant programs for parents with different origins.

More research is warranted to assess at what age unhealthy weight develops in children with immigrant background compared to non-immigrant background children. It is also important to examine changes in weight status from childhood through adolescence and further into adulthood in different immigrant groups, and whether the association between SEP and weight indicators in children with immigrant background will change. Further, there is a need to examine whether factors other than SEP may explain differences in weight status by immigrant background.

## Conclusion

In Norway, children with immigrant background had a higher prevalence of overweight/obesity and WHtR ≥ 0.5 than non-immigrant background children. Children originating from Southern/Eastern Europe, Asia except South-Asia and Africa had higher prevalence of overweight/obesity than non-immigrant background children, and children with immigrant background from Asia except South-Asia had higher prevalence of WHtR ≥ 0.5, regardless of parental education. Culturally acceptable preventative measures targeting the parents of vulnerable immigrant background children before the school-age period should be implemented to prevent development of unhealthy weight status.

### Supplementary Information

**Additional file 1:** **Supplementary Text 1.** Division of children with immigrant background into regional groups

**Additional file 2:** **Supplementary Table 1.** Prevalence of IOTF BMI categories* by children with non-immigrant and immigrant background.

**Additional file 3:** **Supplementary Table 2.** Immigrant category by total and by immigrant and regional background.

**Additional file 4:** **Supplementary Table 3.** Prevalence of overweight/obesity and WHtR ≥ 0.5 by sex within groups.

**Additional file 5:** **Supplementary Table 4.** Prevalence of overweight/obesity and WHtR ≥ 0.5 by parental education levels within groups.

**Additional file 6:** **Supplementary Table 5.** Prevalence ratios of overweight/obesity for immigrant background children with non-immigrants as the reference.

**Additional file 7:** **Supplementary Table 6. **Prevalence ratios of WHtR ≥ 0.5 for immigrant background children with non-immigrants as the reference.

**Additional file 8:** **Supplementary Table 7.** Prevalence of IOTF BMI categories* using the suggested lower cut-offs for individuals originating from South-Asia^a^.

**Additional file 9:** **Supplementary Table 8.** Prevalence ratios of overweight/obesity* using the lower suggested cut-offs for children originating from South-Asia^a^.

**Additional file 10:** **Supplementary Table 9.** Sensitivity analysis providing odds ratios of overweight/obesity and WHtR ≥ 0.5 for immigrant background children.

**Additional file 11:** **Supplementary Table 10.** Prevalence of overweight/obesity and WHtR ≥ 0.5 by foreign- versus Norwegian-born immigrant children.

## Data Availability

The dataset generated and analysed during the current study are available through The Norwegian Growth Cohort from The Norwegian Institute of Public Health after application to helsedata.no: https://www.fhi.no/div/helseundersokelser/vekstkohorten/tilgang-til-data-fra-vekstkohorten/#soek-om-datatilgang (website in Norwegian) or contact vekstkohorten@fhi.no. Information on access for non-Norwegians: https://www.fhi.no/en/more/access-to-data/applying-for-access-to-data.

## Abbreviations

Asia except S-A: Asia except South-Asia
BMI: Body mass index
CI: Confidence interval
GEE: Generalized estimating equation
IOTF: International Obesity Task Force
NCGS: Norwegian Childhood Growth Study
ov/ob: Overweight including obesity (body mass index ≥ 25 kg/m^2^)
PORs: Prevalence odds ratios
PR: Prevalence ratio
SD: Standard deviation
SEP: Socio-economic position
S/E: Southern and Eastern
W/N: Western and Northern
WHtR: Waist to heigh ratio
