# Supplementary material for: The influence of immigrant background and parental education on overweight and obesity in 8-year-old children in Norway
Source: BMC Public Health. 2023 Aug 29;23:1660. doi: 10.1186/s12889-023-16571-1 (PMC10466865; doi:10.1186/s12889-023-16571-1)
Supplement: Supplementary file 1 — Additional file 1: Supplementary Text 1. Division of children with immigrant background into regional groups [file 12889_2023_16571_MOESM1_ESM.docx]

**Supplementary Text 1. Division of children with immigrant background into regional groups**

Due to expected heterogeneity within children with immigrant background, they were split into subgroups by region of origin; mainly based on the categorization according to Statistics Norway. However, European countries were categorized based on existing knowledge about overweight and obesity prevalence among adults with immigrant background in Norway^[[1]](#footnote-2)^ and among children living in some of these countries^[[2]](#footnote-3)^, as well as biological, social, and cultural similarities (and not according to European Union membership as in Statistics Norway’s categories). The World Health Organization Western Pacific Region suggest that overweight and obesity is defined by using lower body mass index criteria in Asian populations^^[[3]](#footnote-4)^^, therefore we divided Asia into two groups (Asia except South-Asia and South-Asia). A separate South-Asian group is also in accordance with similar studies^[[4]](#footnote-5),^^[[5]](#footnote-6),^^[[6]](#footnote-7),^^[[7]](#footnote-8),^^[[8]](#footnote-9)^**.** The countries (n = 87) included in the subgroups by region of origin are:

- *Western and Northern Europe*: Children with origin from Denmark, Finland, Iceland, Sweden, Belgium, Estonia, France, Ireland, Latvia, Netherlands, Lithuania, United Kingdom, and Germany.
- *Southern and Eastern Europe*: Children with origin from Albania, Greece, Belarus, Croatia, Italy, Poland, Portugal, Romania, Spain, Russia, Ukraine, Hungary, Bosnia-Herzegovina, North Macedonia, Slovakia, Czech Republic, Serbia, and Kosovo (Kosovo included here as it previously was a part of Serbia).
- *Asia except South-Asia*: Children with origin from Turkey, Afghanistan, Azerbaijan, Myanmar, Philippines, Hong Kong, Indonesia, Iraq, Iran, Israel, Jordan, Cambodia, Kazakhstan, China, Kyrgyzstan, Lebanon, Malaysia, Mongolia, Palestine, Tajikistan, Turkmenistan, Uzbekistan, Syria, Thailand, and Vietnam.
- *South-Asia*: Children with origin from Bangladesh, Sri Lanka, India, Nepal, and Pakistan.
- *Africa*: Children with origin from Algeria, Angola, Burundi, Côte d’Ivore, Eritrea, Ethiopia, Egypt, Ghana, Cameroon, Kenya, Congo, Lesotho, Liberia, Libya, Madagascar, Malawi, Morocco, Nigeria, Rwanda, Sierra Leone, Somalia, Sudan, Tanzania, Tunisia, Uganda, and Zambia.

1. Kjøllesdal M, Straiton ML, Øien-Ødegaard C, Aambø A, Holmboe O, Johansen R, Grewal NG, Indseth T. "Helse blant innvandrere i Norge" [Health among immigrants in Norway] 2019. Oslo: Folkehelseinstituttet, 2019. [↑](#footnote-ref-2)
2. Spinelli A, Buoncristiano M, Nardone P, Starc G, Hejgaard T, Júlíusson PB, et al. Thinness, overweight, and obesity in 6- to 9-year-old children from 36 countries: The World Health Organization European Childhood Obesity Surveillance Initiative-COSI 2015-2017. Obes Rev. 2021;22 Suppl 6:e13214. [↑](#footnote-ref-3)
3. The World Health Organization. Regional Office for the Western Pacific Region. The Asia-Pacific perspective: Redefining obesity and its treatment. Sydney: Health Communications Australia; 2000. [↑](#footnote-ref-4)
4. Toftemo I, Jenum AK, Lagerløv P, Júlίusson PB, Falk RS, Sletner L. Contrasting patterns of overweight and thinness among preschool children of different ethnic groups in Norway, and relations with maternal and early life factors. BMC Public Health. 2018 Aug 23;18(1):1056. doi: 10.1186/s12889-018-5952-1. PMID: 30139343; PMCID: PMC6108110. [↑](#footnote-ref-5)
5. Hudda MT, Nightingale CM, Donin AS, Fewtrell MS, Haroun D, Lum S, Williams JE, Owen CG, Rudnicka AR, Wells JCK, Cook DG, Whincup PH. Body mass index adjustments to increase the validity of body fatness assessment in UK Black African and South Asian children. Int J Obes (Lond). 2017 Jul;41(7):1048-1055. doi: 10.1038/ijo.2017.75. Epub 2017 Mar 22. PMID: 28325931; PMCID: PMC5500188. [↑](#footnote-ref-6)
6. de Wilde JA, van Dommelen P, Middelkoop BJ. Appropriate body mass index cut-offs to determine thinness, overweight and obesity in South Asian children in the Netherlands. PLoS One. 2013 Dec 19;8(12):e82822. doi: 10.1371/journal.pone.0082822. PMID: 24367559; PMCID: PMC3868582. [↑](#footnote-ref-7)
7. Jenum AK, Diep LM, Holmboe-Ottesen G, Holme IM, Kumar BN, Birkeland KI. Diabetes susceptibility in ethnic minority groups from Turkey, Vietnam, Sri Lanka and Pakistan compared with Norwegians - the association with adiposity is strongest for ethnic minority women. BMC Public Health. 2012 Mar 1;12:150. doi: 10.1186/1471-2458-12-150. PMID: 22380873; PMCID: PMC3315409. [↑](#footnote-ref-8)
8. Nightingale CM, Rudnicka AR, Owen CG, Cook DG, Whincup PH. Patterns of body size and adiposity among UK children of South Asian, black African-Caribbean and white European origin: Child Heart And health Study in England (CHASE Study). Int J Epidemiol. 2011 Feb;40(1):33-44. doi: 10.1093/ije/dyq180. Epub 2010 Nov 1. PMID: 21044977; PMCID: PMC3043281. [↑](#footnote-ref-9)
