# Supplementary material for: The influence of immigrant background and parental education on overweight and obesity in 8-year-old children in Norway
Source: BMC Public Health. 2023 Aug 29;23:1660. doi: 10.1186/s12889-023-16571-1 (PMC10466865; doi:10.1186/s12889-023-16571-1)
Supplement: Supplementary file 2 — Additional file 2: Supplementary Table 1. Prevalence of IOTF BMI categories* by children with non-immigrant and immigrant background. [file 12889_2023_16571_MOESM2_ESM.docx]

**Supplementary Table 1. Prevalence of IOTF BMI categories* by children with non-immigrant and immigrant background (n = 8858).**

|  | Non-immigrant background (n = 7575) | Immigrant background, total  (n = 1283) | Immigrant background, by region of origin | | | | |
| --- | --- | --- | --- | --- | --- | --- | --- |
|  |  |  | Western and Northern Europe (n = 142) | Southern and Eastern Europe (n = 288) | Asia except South-Asia  (n = 449) | South-Asia  (n = 181) | Africa  (n = 223) |
| Thinness | 7.8 (7.2, 8.4) | 11.0 (9.4, 12.8) | 12.7 (8.1, 19.3) | 5.6 (3.4, 8.9) | 10.0 (7.6, 13.2) | 17.1 (12.3, 2.8) | 13.9 (9.9, 19.1) |
| Normal | 76.2 (75.2, 77.1) | 68.5 (65.9, 71.0) | 73.9 (66.0, 80.6) | 72.2 (66.7, 77.1) | 69.3 (64.8, 73.4) | 64.6 (57.4, 71.3) | 61.9 (55.3, 68.1) |
| Overweight | 12.9 (12.2, 13.7) | 15.3 (13.4, 17.4) | 9.9 (5.9, 16.0) | 15.6 (11.9, 20.3) | 14.5 (11.5, 18.1) | 15.5 (10.9, 21.5) | 19.7 (15.0, 25.5) |
| Obesity | 3.1 (2.8, 3.5) | 5.2 (4.1, 6.6) | 3.5 (1.5, 8.2) | 6.6 (4.2, 10.1) | 6.2 (4.3, 8.9) | 2.8 (1.2, 6.5) | 4.5 (2.4, 8.2) |
| Prevalence of IOTF BMI categories* by children with non-immigrant and immigrant background in total, and groups by region of origin (n = 8,858). Numbers show percent and 95% confidence intervals.  * Age- and sex-specific BMI cut-off-values based on the International Obesity Task Force (IOTF) criteria^[[1]](#footnote-2)^.  BMI: body mass index; n: number; IOTF: International Obesity Task Force. | | | | | | | |

1. Cole TJ, Lobstein T. Extended international (IOTF) body mass index cut-offs for thinness, overweight and obesity. Pediatr Obes. 2012;7(4):284-94. [↑](#footnote-ref-2)
