# Supplementary material for: The influence of immigrant background and parental education on overweight and obesity in 8-year-old children in Norway
Source: BMC Public Health. 2023 Aug 29;23:1660. doi: 10.1186/s12889-023-16571-1 (PMC10466865; doi:10.1186/s12889-023-16571-1)
Supplement: Supplementary file 3 — Additional file 3: Supplementary Table 2. Immigrant category by total and by immigrant and regional background. [file 12889_2023_16571_MOESM3_ESM.docx]

**Supplementary Table 2. Immigrant category by total and by immigrant and regional background.**

|  | Total  (n = 8858) | Non-immigrant background  (n = 7575) | Immigrant background, total  (n = 1283) | Immigrant background, by region of origin | | | | |
| --- | --- | --- | --- | --- | --- | --- | --- | --- |
|  |  |  |  | Western and Northern Europe  (n = 142) | Southern and Eastern Europe  (n = 288) | Asia except South-Asia (n = 449) | South-Asia  (n = 181) | Africa  (n = 223) |
|  |  |  |  |  |  |  |  |  |
| Norwegian-born to Norwegian-born parents | 84.3  (7471) | 98.6  (7471) |  |  |  |  |  |  |
| Foreign-born to Norwegian-born parents | 1.2  (104) | 1.4  (104) |  |  |  |  |  |  |
| Immigrants | 5.7  (502) |  | 39.1  (502) | 73.2  (104) | 58.3 (168) | 27.2  (122) | 8.3  (15) | 41.7  (93) |
| Norwegian-born to immigrant parents | 8.8  (781) |  | 60.9  (781) | 26.8  (38) | 41.7  (120) | 72.8  (327) | 91.7  (166) | 58.3  (130) |
|  |  |  |  |  |  |  |  |  |
| Details of immigrant category, % (n), by children with non-immigrant and immigrant background in total, and groups by region of origin (n = 8858). | | | | | | | | |
