# Supplementary material for: The influence of immigrant background and parental education on overweight and obesity in 8-year-old children in Norway
Source: BMC Public Health. 2023 Aug 29;23:1660. doi: 10.1186/s12889-023-16571-1 (PMC10466865; doi:10.1186/s12889-023-16571-1)
Supplement: Supplementary file 4 — Additional file 4: Supplementary Table 3. Prevalence of overweight/obesity and WHtR ≥ 0.5 by sex within groups. [file 12889_2023_16571_MOESM4_ESM.docx]

**Supplementary Table 3. Prevalence of overweight/obesity and WHtR ≥ 0.5 by sex within groups.**

|  | Non-immigrant background  (n = 7575^a^) | | Immigrant background, total  (n = 1283) | | Immigrant background, by region of origin | | | | | | | | | | |
| --- | --- | --- | --- | --- | --- | --- | --- | --- | --- | --- | --- | --- | --- | --- | --- |
|  |  | |  | | Western and  Northern Europe  (n = 142) | | Southern and  Eastern Europe (n = 288) | | Asia except  South-Asia (n = 449) | | South-Asia  (n = 181) | | | Africa (n = 223) | |
|  | Boys  (n = 3927) | Girls  (n = 3648) | Boys  (n = 615) | Girls  (n = 668) | Boys  (n = 83) | Girls  (n = 59) | Boys  (n = 138) | Girls  (n = 150) | Boys  (n = 208) | Girls  (n = 241) | Boys  (n = 93) | Girls  (n = 88) | Boys  (n = 93) | | Girls  (n = 130) |
| Ov/ob | 14.0  (13.0, 15.2) | 18.2  (17.0, 19.5) | 18.7  (15.8, 22.0) | 22.2  (19.2, 25.5) | 7.2  (3.3, 15.3) | 22.0  (13.2, 34.5) | 21.0  (15.0, 28.6) | 23.3  (17.2, 30.8) | 19.2  (14.4, 25.2) | 22.0  (17.2, 27.7) | 19.4  (12.5, 28.7) | 17.0  (10.5, 26.4) | 23.7  (16.1, 33.4) | | 24.6  (17.9, 32.8) |
| Norm/thin | 86.0  (84.9, 87.0) | 81.8  (80.5, 83.0) | 81.3  (78.0, 84.2) | 77.8  (74.5, 80.8) | 92.8  (84.7, 96.7) | 78.0  (65.5, 86.8) | 79.0  (71.4, 85.0) | 76.7  (69.2, 82.8) | 80.8  (74.8, 85.6) | 78.0  (72.3, 82.8) | 80.7  (71.3, 87.5) | 83.0  (73.6, 89.5) | 76.3  (66.2, 83.9) | | 75.4  (67.2, 82.1) |
| p-value | <0.001 | | 0.125 | | 0.011 | | 0.636 | | 0.472 | | 0.688 | | 0.869 | | |
|  |  |  |  |  |  |  |  |  |  |  |  |  |  | |  |
| WHtR ≥ 0.5 | 6.8  (6.1, 7.7) | 8.5  (7.7, 9.5) | 12.4  (10.0, 15.2) | 10.2  (8.1, 12.7) | 4.8  (1.8, 12.4) | 8.5  (3.5, 18.9) | 13.0 (8.4, 19.8) | 9.3  (5.6, 15.2) | 13.9 (9.9, 19.4) | 12.5  (8.8, 17.3) | 14.0  (8.3, 22.7) | 10.2  (5.4, 18.6) | 12.9  (7.5, 21.4) | | 7.7  (4.2, 13.8) |
| WHtR < 0.5 | 93.2  (92.3, 93.9) | 91.5  (90.5, 92.4) | 87.6  (84.8, 90.0) | 89.8  (87.3, 91.9) | 95.2  (87.8, 98.2) | 91.5  (81.1, 98.2) | 87.0  (80.2, 91.6) | 90.7  (84.8, 94.4) | 86.1  (80.6, 90.2) | 87.6  (82.7, 91.2) | 86.0  (77.2, 91.7) | 89.8  (81.4, 94.6) | 87.1  (78.6, 92.6) | | 92.3  (86.3, 95.8) |
| p-value | 0.006 | | 0.217 | | 0.490^b^ | | 0.317 | | 0.640 | | 0.440 | | 0.198 | | |
| Prevalence of overweight/obesity and WHtR ≥ 0.5 by sex within children with non-immigrant and immigrant background in total, and groups by region of origin. Numbers show percent and 95% confidence intervals. X^2^-tests or Fishers exact tests were conducted for differences between boys and girls within groups.  ^a^ n for WHtR: 7566; 3923 boys and 3643 girls. ^b^ p-value with Fishers exact due to low n. n: number; norm/thin: normal or thin ; ov/ob: overweight including obesity ; WHtR: waist-to-heigh-ratio. | | | | | | | | | | | | | | | |
