# Supplementary material for: The influence of immigrant background and parental education on overweight and obesity in 8-year-old children in Norway
Source: BMC Public Health. 2023 Aug 29;23:1660. doi: 10.1186/s12889-023-16571-1 (PMC10466865; doi:10.1186/s12889-023-16571-1)
Supplement: Supplementary file 5 — Additional file 5: Supplementary Table 4. Prevalence of overweight/obesity and WHtR ≥ 0.5 by parental education levels within groups. [file 12889_2023_16571_MOESM5_ESM.docx]

**Supplementary Table 4. Prevalence of overweight/obesity and WHtR ≥ 0.5 by parental education levels within groups.**

|  | Non-immigrant background  (n = 7573)^a^ | | | Immigrant background, total (n = 1238) | | | Immigrant background, by region of origin | | | | | | | | | | | | | | |
| --- | --- | --- | --- | --- | --- | --- | --- | --- | --- | --- | --- | --- | --- | --- | --- | --- | --- | --- | --- | --- | --- |
|  |  | | |  | | | Western and Northern Europe (n = 131) | | | Southern and Eastern Europe (n = 283) | | | Asia except South-Asia (n = 434) | | | South-Asia (n = 178) | | | Africa  (n = 212) | | |
|  | Pri  (n = 301) | Sec  (n= 2548) | Hi  (n = 4724) | Pri  (n = 357) | Sec  (n = 413) | Hi  (n = 468) | Pri  (n = 13) | Sec  (n = 30) | Hi  (n = 88) | Pri  (n = 45) | Sec  (n = 121) | Hi  (n = 117) | Pri  (n = 139) | Sec  (n = 147) | Hi  (n = 148) | Pri  (n = 64) | Sec  (n = 66) | Hi  (n = 48) | Pri  (n = 96) | Sec  (n = 49) | Hi  (n = 67) |
| Ov/ob | 18.9  (14.9,  23.8) | 19.1  (17.6,  20.7) | 14.2  (13.2,  15.2) | 19.3  (15.6,  23.8) | 23.5  (19.6,  27.8) | 18.0  (14.7,  21.7) | 15.4 (3.8, 45.4) | 16.7 (7.1, 34.5) | 8.0  (3.8, 15.9) | 22.2 (12.4, 36.7) | 28.9  (21.5,  37.7) | 16.2  (10.6,  24.1) | 17.3  (11.8,  24.5) | 22.5  (16.4,  29.9) | 23.0  (16.9,  30.5) | 18.8 (10.9, 30.3) | 16.7 (9.4, 27.7) | 16.7 (8.5, 30.1) | 21.9 (14.7, 31.3) | 26.5 (16.0, 40.6) | 28.9 (15.1, 35.6) |
| Norm/  thin | 81.1  (76.2,  85.1) | 80.9  (79.4,  82.4) | 85.8  (84.8,  86.8) | 80.7  (76.2,  84.5) | 76.5  (72.2,  80.4) | 82.1  (78.3,  85.3) | 84.6 (54.6, 96.2) | 83.3 (65.5, 93.0) | 92.1 (84.2, 96.2) | 77.8 (63.3, 87.6) | 71.1  (62.4,  78.5) | 83.8  (75.9,  89.4) | 82.7  (75.5,  88.2) | 77.6  (70.1,  83.6) | 77.0  (69.6,  83.1) | 81.3 (69.7, 89.1) | 83.3 (72.3, 90.6) | 83.3 (70.0, 91.5) | 78.1 (68.7, 85.3) | 73.5 (59.4, 84.0) | 76.1 (64.4, 84.9) |
| p-value | <0.001 | | | 0.110 | | | 0.208^b^ | | | 0.065 | | | 0.427 | | | 0.940 | | | 0.821 | | |
|  |  |  |  |  |  |  |  |  |  |  |  |  |  |  |  |  |  |  |  |  |  |
| WHtR ≥0.5 | 11.6  (8.5,  15.8) | 10.3  (9.2,  11.5) | 5.9  (5.3,  6.7) | 12.0  (9.1,  15.9) | 13.1  (10.2,  16.7) | 8.3  (6.2,  11.2) | 15.4 (3.8, 45.4) | 6.7  (1.7, 23.3) | 2.3  (0.6,  8.7) | 13.3 (6.1, 26.7) | 14.1  (8.9,  21.5) | 7.7  (4.0,  14.2) | 13.0  (8.3,  19.6) | 12.2  (7.8,  18.6) | 14.9  (10.0,  21.6) | 10.9 (5.3, 21.3) | 15.2 (8.3, 26.0) | 6.3  (2.0, 17.8) | 10.4 (5.7, 18.4) | 14.3 (6.9, 27.2) | 4.5  (1.4, 13.1) |
| WHtR <0.5 | 88.4  (84.2,  91.5) | 89.7  (88.5,  90.8) | 94.1  (93.4,  94.7) | 88.0  (84.2,  91.0) | 86.9  (83.3,  89.9) | 91.7  (88.8,  93.9) | 84.6 (54.6, 96.2) | 93.3 (76.7, 98.4) | 97.7 (91.3, 99.4) | 86.7 (73.3, 93.9) | 86.0  (78.5,  91.1) | 92.3  (85.8,  96.0) | 87.1  (80.4,  91.7) | 87.8  (81.4,  92.2) | 85.1  (78.4,  90.0) | 89.1 (78.7, 94.7) | 84.9 (74.0, 91.7) | 93.8 (82.2, 98.0) | 89.6 (81.7, 94.3) | 85.7 (72.9, 93.1) | 95.5 (86.9, 98.6) |
| p-value | <0.001 | | | 0.060 | | | 0.063^b^ | | | 0.270 | | | 0.792 | | | 0.343^b^ | | | 0.177^b^ | | |
| Prevalence of overweight/obesity and WHtR ≥ 0.5 by parental education levels within children with non-immigrant and immigrant background in total, and groups by region of origin. Numbers show percent and 95% confidence intervals. X^2^-tests or Fishers exact tests were conducted for differences between parental education levels within groups. Highest parental education level by either mother or father attained the year of measurement: primary (primary education or less), secondary (lower and upper secondary education), and higher education (education in university/college).  ^a^ n for WHtR: 7564. 301 with primary, 2545 with secondary and 4718 with higher education. ^b^ p-value with Fishers exact due to low n. Hi: higher; n: number; norm/thin: normal or thin ; ov/ob: overweight including obesity ; pri: primary; sec: secondary; WHtR: waist-to-heigh-ratio. | | | | | | | | | | | | | | | | | | | | | |
