# Supplementary material for: The influence of immigrant background and parental education on overweight and obesity in 8-year-old children in Norway
Source: BMC Public Health. 2023 Aug 29;23:1660. doi: 10.1186/s12889-023-16571-1 (PMC10466865; doi:10.1186/s12889-023-16571-1)
Supplement: Supplementary file 6 — Additional file 6: Supplementary Table 5. Prevalence ratios of overweight/obesity for immigrant background children with non-immigrants as the reference. [file 12889_2023_16571_MOESM6_ESM.docx]

**Supplementary Table 5. Prevalence ratios of overweight/obesity for immigrant background children with non-immigrants as the reference.**

|  | Model 1 | | Model 2 | | Model 3 | |
| --- | --- | --- | --- | --- | --- | --- |
|  | PR (95% CI) | p-value | PR (95% CI) | p-value | PR (95% CI) | p-value |
| Non-immigrant background | Reference |  | Reference |  | Reference |  |
| Immigrant background, total | 1.24 (1.09 – 1.41) | 0.001 | 1.28 (1.12 – 1.45) | <0.001 | 1.24 (1.08 – 1.41) | 0.002 |
|  |  |  |  |  |  |  |
| Non-immigrant background | Reference |  | Reference |  | Reference |  |
| Western and Northern Europe | 0.70 (0.43 – 1.14) | 0.149 | 0.69 (0.42 – 1.12) | 0.134 | 0.70 (0.43 – 1.15) | 0.155 |
| Southern and Eastern Europe | 1.39 (1.12 – 1.73) | 0.003 | 1.42 (1.14 – 1.78) | 0.002 | 1.37 (1.10 – 1.72) | 0.005 |
| Asia except South-Asia | 1.27 (1.05 – 1.54) | 0.014 | 1.32 (1.09 – 1.61) | 0.005 | 1.28 (1.05 – 1.57) | 0.016 |
| South-Asia | 1.04 (0.74 – 1.46) | 0.820 | 1.11 (0.79 – 1.56) | 0.563 | 1.06 (0.75 – 1.50) | 0.745 |
| Africa | 1.44 (1.12 – 1.85) | 0.004 | 1.51 (1.17 – 1.94) | 0.001 | 1.47 (1.13 – 1.91) | 0.004 |
| Prevalence ratios (PR) (95% CI) of overweight/obesity for 8-year-old children (n = 8,669) in Norway by immigrant background and groups by region of origin. Three sets of GEE log-binominal models were conducted using children with non-immigrant background as the reference category. Model 1 with adjustments for age, sex, and survey year; model 2 additionally adjust for residing area and population density; and model 3 additionally adjust for parental education level. The analyses were conducted with complete cases on all covariates. CI: confidence interval; GEE: generalized estimating equation; n: number; ov/ob: overweight including obesity; PR: Prevalence ratio; WHtR: waist-to-heigh-ratio. | | | | | | |
