# Supplementary material for: The influence of immigrant background and parental education on overweight and obesity in 8-year-old children in Norway
Source: BMC Public Health. 2023 Aug 29;23:1660. doi: 10.1186/s12889-023-16571-1 (PMC10466865; doi:10.1186/s12889-023-16571-1)
Supplement: Supplementary file 7 — Additional file 7: Supplementary Table 6. Prevalence ratios of WHtR ≥ 0.5 for immigrant background children with non-immigrants as the reference. [file 12889_2023_16571_MOESM7_ESM.docx]

**Supplementary Table 6. Prevalence ratios of WHtR ≥ 0.5 for immigrant background children with non-immigrants as the reference.**

|  | Model 1 | | Model 2 | | Model 3 | |
| --- | --- | --- | --- | --- | --- | --- |
|  | PR (95% CI) | p-value | PR (95% CI) | p-value | PR (95% CI) | p-value |
| Non-immigrant background | Reference |  | Reference |  | Reference |  |
| Immigrant background, total | 1.45 (1.21 – 1.74) | <0.001 | 1.52 (1.26 – 1.82) | <0.001 | 1.35 (1.10 – 1.64) | 0.003 |
|  |  |  |  |  |  |  |
| Non-immigrant background | Reference |  | Reference |  | Reference |  |
| Western and Northern Europe | 0.63 (0.30 – 1.35) | 0.236 | 0.62(0.29 – 1.34) | 0.224 | 0.63 (0.29 – 1.36) | 0.241 |
| Southern and Eastern Europe | 1.47 (1.06 – 2.04) | 0.021 | 1.51 (1.08 – 2.11) | 0.015 | 1.38 (0.99 – 1.93) | 0.060 |
| Asia except South-Asia | 1.77 (1.38 – 2.27) | <0.001 | 1.89 (1.46 – 2.44) | <0.001 | 1.64 (1.25 – 2.15) | <0.001 |
| South-Asia | 1.49 (0.98 – 2.27) | 0.063 | 1.63 (1.05 – 2.52) | 0.028 | 1.38 (0.89 – 2.15) | 0.154 |
| Africa | 1.26 (0.83 – 1.91) | 0.270 | 1.35 (0.88 – 2.06) | 0.167 | 1.17 (0.76 – 1.82) | 0.478 |
| Prevalence ratios (PR) (95% CI) of WHtR ≥ 0.5 for 8-year-old children (n = 8660) in Norway by immigrant background and groups by region of origin. Three sets of GEE log-binominal models were conducted using children with non-immigrant background as the reference category. Model 1 with adjustments for age, sex, and survey year; model 2 additionally adjust for residing area and population density; and model 3 additionally adjust for parental education level. The analyses were conducted with complete cases on all covariates. CI: confidence interval; GEE: generalized estimating equation; n: number; ov/ob: overweight including obesity; PR: Prevalence ratio; WHtR: waist-to-heigh-ratio. | | | | | | |
