# Supplementary material for: The influence of immigrant background and parental education on overweight and obesity in 8-year-old children in Norway
Source: BMC Public Health. 2023 Aug 29;23:1660. doi: 10.1186/s12889-023-16571-1 (PMC10466865; doi:10.1186/s12889-023-16571-1)
Supplement: Supplementary file 8 — Additional file 8: Supplementary Table 7. Prevalence of IOTF BMI categories* using the suggested lower cut-offs for individuals originating from South-Asiaa. [file 12889_2023_16571_MOESM8_ESM.docx]

**Supplementary Table 7. Prevalence of IOTF BMI categories* using the suggested lower cut-offs for individuals originating from South-Asia^a^.**

|  | Non-immigrant background  (n = 7575) | Immigrant background, total  (n = 1283) | Immigrant background, total^b^ (n = 1283) | Change when using lower  cut-offs, % | South-Asia  (n = 181) | South-Asia^b^ (n = 181) | Change when using lower  cut-offs, % |
| --- | --- | --- | --- | --- | --- | --- | --- |
|  |  |  |  |  |  |  |  |
| Thinness | 7.8 (7.2, 8.4) | 11.0 (9.4, 12.8) | 11.0 (9.4, 12.8) | 0 | 17.1 (12.3, 2.8) | 17.1 (12.3, 23.3) | 0 |
| Normal | 76.2 (75.2, 77.1) | 68.5 (65.9, 71.0) | 66.6 (63.9, 69.1) | -1.9 | 64.6 (57.4, 71.3) | 50.8 (43.6, 58.0) | -13.8 |
| Overweight | 12.9 (12.2, 13.7) | 15.3 (13.4, 17.4) | 16.1 (14.2, 18.2) | +0.8 | 15.5 (10.9, 21.5) | 21.0 (15.7, 27.5) | +5.5 |
| Obesity | 3.1(2.8, 3.5) | 5.2 (4.1, 6.6) | 6.4 (5.2, 7.9) | +1.2 | 2.8 (1.2, 6.5) | 11.1 (7.2, 16.5) | +8.3 |
|  |  |  |  |  |  |  |  |
| Ov/ob | 16.0 (15.2, 16.9) | 20.5 (18.4, 22.8) | 22.5 (15.2, 16.9) | +2.0 | 18.2 (13.3, 24.5) | 32.0 (25.7, 39.2) | +13.8 |
| Norm/thin | 84.0 (83.1, 84.8) | 79.5 (77.2, 81.6) | 77.6 (75.2, 79.8) | -2.0 | 81.8 (75.5, 86.7) | 68.0 (60.8, 74.3) | -13.8 |
| Prevalence of IOTF BMI categories* by children with non-immigrant and immigrant background total, also using the suggested lower cut-offs for individuals originating from South-Asia^a^. Numbers show percent and 95% confidence intervals.  * Age- and sex-specific BMI cut-off-values based on the International Obesity Task Force (IOTF) criteria^[[1]](#footnote-2)^.  ^a^ Lower cut-offs for overweight and obesity ^[[2]](#footnote-3)^,^[[3]](#footnote-4)^. ^b^ Group where the lower suggested regional cut-offs for South-Asians are used. BMI: body mass index; IOTF: International Obesity Task Force; norm/thin: normal or thin; ov/ob: overweight including obesity. | | | | | | | |

1. Cole TJ, Lobstein T. Extended international (IOTF) body mass index cut-offs for thinness, overweight and obesity. Pediatr Obes. 2012;7(4):284-94. [↑](#footnote-ref-2)
2. The World Health Organization. Regional Office for the Western Pacific Region. The Asia-Pacific perspective: Redefining obesity and its treatment. Sydney: Health Communications Australia; 2000. & Obesity Classification: World Obesity; [cited 2022 May 13]. Available from: <https://www.worldobesity.org/about/about-obesity/obesity-classification>. [↑](#footnote-ref-3)
3. The World Health Organization. Regional Office for the Western Pacific Region. The Asia-Pacific perspective: Redefining obesity and its treatment. Sydney: Health Communications Australia; 2000. & Obesity Classification: World Obesity; [cited 2022 May 13]. Available from: <https://www.worldobesity.org/about/about-obesity/obesity-classification>. [↑](#footnote-ref-4)
