# Supplementary material for: The influence of immigrant background and parental education on overweight and obesity in 8-year-old children in Norway
Source: BMC Public Health. 2023 Aug 29;23:1660. doi: 10.1186/s12889-023-16571-1 (PMC10466865; doi:10.1186/s12889-023-16571-1)
Supplement: Supplementary file 9 — Additional file 9: Supplementary Table 8. Prevalence ratios of overweight/obesity* using the lower suggested cut-offs for children originating from South-Asiaa. [file 12889_2023_16571_MOESM9_ESM.docx]

**Supplementary Table 8. Prevalence ratios of overweight/obesity* using the lower suggested cut-offs for children originating from South-Asia^a^.**

|  | Model 1 | | Model 2 | | Model 3 | |
| --- | --- | --- | --- | --- | --- | --- |
|  | PR (95% CI) | p-value | PR (95% CI) | p-value | PR (95% CI) | p-value |
| Non-immigrant background | 1.00 |  | 1.00 |  | 1.00 |  |
| Immigrant background, total^b^ | 1.35 (1.20 – 1.52) | <0.001 | 1.39 (1.23 – 1.57) | <0.001 | 1.35 (1.19 – 1.54) | <0.001 |
|  |  |  |  |  |  |  |
| Non-immigrant background | 1.00 |  | 1.00 |  | 1.00 |  |
| South-Asia^b^ | 1.90 (1.51 – 2.40) | <0.001 | 2.03 (1.60 – 2.57) | <0.001 | 1.97 (1.55 – 2.51) | <0.001 |
| Prevalence ratios (PR) (95% CI) of overweight/obesity* for 8-year-old children in Norway by immigrant background total and individuals originating from South Asia using the lower suggested cut-offs for children originating from South-Asia^a^ (n = 8669). Three sets of GEE log-binominal models were conducted using children with non-immigrant background as the reference category. Model 1 with adjustments for age, sex, and survey year; model 2 additionally adjust for residing area and population density; and model 3 additionally adjust for parental education level. The analyses were conducted with complete cases on all covariates.  * Age- and sex-specific BMI cut-off-values based on the International Obesity Task Force (IOTF) criteria^[[1]](#footnote-2)^.  ^a^ Lower cut-offs for overweight including obesity ^[[2]](#footnote-3)^,^[[3]](#footnote-4)^. ^b^ Group where the lower suggested regional cut-offs for South-Asians are used.  BMI: body mass index; CI: confidence interval; GEE: generalized estimating equation; IOTF: International Obesity Task Force; n: number; ov/ob: overweight including obesity; PR: Prevalence ratio. | | | | | | |

1. Cole TJ, Lobstein T. Extended international (IOTF) body mass index cut-offs for thinness, overweight and obesity. Pediatr Obes. 2012;7(4):284-94. [↑](#footnote-ref-2)
2. The World Health Organization. Regional Office for the Western Pacific Region. The Asia-Pacific perspective: Redefining obesity and its treatment. Sydney: Health Communications Australia; 2000. & Obesity Classification: World Obesity; [cited 2022 May 13]. Available from: <https://www.worldobesity.org/about/about-obesity/obesity-classification>. [↑](#footnote-ref-3)
3. The World Health Organization. Regional Office for the Western Pacific Region. The Asia-Pacific perspective: Redefining obesity and its treatment. Sydney: Health Communications Australia; 2000. & Obesity Classification: World Obesity; [cited 2022 May 13]. Available from: <https://www.worldobesity.org/about/about-obesity/obesity-classification>. [↑](#footnote-ref-4)
