# Supplementary material for: The influence of immigrant background and parental education on overweight and obesity in 8-year-old children in Norway
Source: BMC Public Health. 2023 Aug 29;23:1660. doi: 10.1186/s12889-023-16571-1 (PMC10466865; doi:10.1186/s12889-023-16571-1)
Supplement: Supplementary file 10 — Additional file 10: Supplementary Table 9. Sensitivity analysis providing odds ratios of overweight/obesity and WHtR ≥ 0.5 for immigrant background children. [file 12889_2023_16571_MOESM10_ESM.docx]

**Supplementary Table 9. Sensitivity analysis providing odds ratios of overweight/obesity and WHtR ≥ 0.5 for immigrant background children.**

|  | Model 1 (n = 8669) | | Model 2 (n = 8669) | | Model 3 (n = 8669) | |
| --- | --- | --- | --- | --- | --- | --- |
| Overweight/obesity | OR (95% CI) | p-value | OR (95% CI) | p-value | OR (95% CI) | p-value |
| Non-immigrant background | Reference |  | Reference |  | Reference |  |
| Immigrant background, total | 1.31 (1.12 – 1.54) | 0.001 | 1.37 (1.17 – 1.61) | <0.001 | 1.31 (1.11 – 1.55) | 0.002 |
|  |  |  |  |  |  |  |
| Non-immigrant background | Reference |  | Reference |  | Reference |  |
| Western and Northern Europe | 0.65 (0.38 – 1.14) | 0.134 | 0.65 (0.37 – 1.13) | 0.127 | 0.66 (0.38 – 1.16) | 0.148 |
| Southern and Eastern Europe | 1.53 (1.15 – 2.03) | 0.003 | 1.58 (1.18 – 2.10) | 0.002 | 1.51 (1.13 – 2.01) | 0.005 |
| Asia except South-Asia | 1.36 (1.06 – 1.73) | 0.014 | 1.43 (1.12 – 1.83) | 0.004 | 1.38 (1.07 – 1.77) | 0.014 |
| South-Asia | 1.06 (0.70 – 1.60) | 0.780 | 1.15 (0.76 – 1.73) | 0.519 | 1.08 (0.71 – 1.64) | 0.714 |
| Africa | 1.60 (1.15 – 2.22) | 0.005 | 1.70 (1.22 – 2.36) | 0.002 | 1.64 (1.17 – 2.32) | 0.004 |
|  |  |  |  |  |  |  |
|  | Model 1 (n = 8660) | | Model 2 (n = 8660) | | Model 3 (n = 8660) | |
| WHtR **≥** 0.5 | OR (95% CI) | p-value | OR (95% CI) | p-value | OR (95% CI) | p-value |
| Non-immigrant background | Reference |  | Reference |  | Reference |  |
| Immigrant background, total | 1.52 (1.24 – 1.86) | <0.001 | 1.61 (1.31 – 1.98) | <0.001 | 1.41 (1.13 – 1.76) | 0.002 |
|  |  |  |  |  |  |  |
| Non-immigrant background | Reference |  | Reference |  | Reference |  |
| Western and Northern Europe | 0.62 (0.28 – 1.36) | 0.231 | 0.61 (0.27 – 1.36) | 0.223 | 0.61 (0.27 – 1.38) | 0.237 |
| Southern and Eastern Europe | 1.56 (1.08 – 2.25) | 0.019 | 1.62 (1.11 – 2.36) | 0.013 | 1.46 (1.00 – 2.13) | 0.052 |
| Asia except South-Asia | 1.92 (1.44 – 2.56) | <0.001 | 2.06 (1.53 – 2.77) | <0.001 | 1.78 (1.30 – 2.43) | <0.001 |
| South-Asia | 1.57 (0.97 – 2.53) | 0.064 | 1.73 (1.06 – 2.83) | 0.029 | 1.43 (0.87 – 2.37) | 0.163 |
| Africa | 1.30 (0.82 – 2.07) | 0.260 | 1.40 (0.87 – 2.24) | 0.162 | 1.19 (0.73 – 1.94) | 0.491 |
| Sensitivity analysis providing odds ratios (OR) (95% CI) of overweight/obesity and WHtR ≥ 0.5 for 8-year-old children in Norway by children with immigrant background and groups by region of origin. Three sets of GEE logistic models were conducted using children with non-immigrant background as the reference category. Model 1 with adjustments for age, sex, and survey year; model 2 additionally adjust for residing area and population density; and model 3 additionally adjust for parental education level. The analyses were conducted with complete cases on all covariates.  CI: confidence interval; GEE: generalized estimating equation; n: number; OR: odds ratio; overweight/obesity: overweight including obesity; PR: Prevalence ratio; WHtR: waist-to-heigh-ratio. | | | | | | |
