# Supplementary material for: The influence of immigrant background and parental education on overweight and obesity in 8-year-old children in Norway
Source: BMC Public Health. 2023 Aug 29;23:1660. doi: 10.1186/s12889-023-16571-1 (PMC10466865; doi:10.1186/s12889-023-16571-1)
Supplement: Supplementary file 11 — Additional file 11: Supplementary Table 10. Prevalence of overweight/obesity and WHtR ≥ 0.5 by foreign- versus Norwegian-born immigrant children. [file 12889_2023_16571_MOESM11_ESM.docx]

**Supplementary Table 10. Prevalence of overweight/obesity and WHtR ≥ 0.5 by foreign- versus Norwegian-born immigrant children.**

|  | Foreign-born immigrant background  (n = 502) | Norwegian-born immigrant background  (n = 781) |
| --- | --- | --- |
| Ov/ob | 21.9 (18.5, 25.8) | 19.6 (17.0, 22.5) |
| Norm/thin | 78.1 (74.3, 81.5) | 80.4 (77.5, 83.1) |
| p-value | 0.315 | |
|  |  |  |
| WHtR ≥ 0.5 | 11.8 (9.2, 14.9) | 10.9 (8.9, 13.3) |
| WHtR < 0.5 | 88.3 (85.1, 90.8) | 89.1 (86.7, 91.1) |
| p-value | 0.630 | |
| Prevalence of overweight/obesity and WHtR ≥ 0.5 by foreign- versus Norwegian-born immigrant children (n = 1283). Numbers show percent and 95% confidence intervals. X^2^-tests were conducted for differences between foreign- versus Norwegian-born immigrant background children.  n: number; norm/thin: normal or thin; ov/ob: overweight including obesity; SD: Standard deviation; WHtR: waist-to-heigh-ratio. | | |
